# Oncocytic Carcinoma of the Cranio-Orbital Area: A Case Report

**DOI:** 10.3389/fsurg.2022.897319

**Published:** 2022-07-01

**Authors:** Peng Yang, Libin Jiang, Honggang Liu, Jialiang Zhang, Jun Kang

**Affiliations:** ^1^ Department of Neurosurgery, Beijing Tongren Hospital, Capital Medical University, Beijing, China; ^2^ Department of Ophthalmology, Beijing Tongren Hospital, Capital Medical University, Beijing, China; ^3^ Department of Pathology, Beijing Tongren Hospital, Capital Medical University, Beijing, China

**Keywords:** oncocytic carcinoma, craniotomy, cranio-orbital, paranasal sinus, orbital

## Abstract

Oncocytic carcinoma is a malignant tumor characterized by a proliferation of epithelial cells with abundant eosinophilic granular cytoplasm. In this article, we report on the first case of a 61-year-old male patient presenting with oncocytic carcinoma involving the cranio-orbital area. An oncocytic carcinoma in the patient, who reported a sudden decrease in vision in his right eye, was removed through a frontal orbital approach craniotomy. The patient's postoperative development was rapid, and he was admitted to the neurosurgery department for a combined operation after ophthalmological screening. Pathological analysis revealed the tumour cells were large, round or polygonal, and the cytoplasm was finely granular and appeared to be more pleomorphic than the eosinophilic adenoma. Oncocytic carcinoma in the cranio-orbital area is extremely rare. The most effective treatment is early resection to be performed jointly by ophthalmology and neurosurgery, and long-term follow-up and adjuvant chemoradiotherapy are beneficial.

## Introduction

Oncocytic carcinoma is also known as oncocytic adenocarcinoma, malignant oncocytoma or malignant oncocytic adenoma. It is a malignant tumor characterized by a proliferation of epithelial cells with abundant eosinophilic granular cytoplasm. Oncocytic carcinoma from a pleomorphic adenoma can be primary or malignant. The majority (80%) of oncocytic carcinomas occur in the parotid gland and 10% occur in the submandibular gland. The rest occur in the small salivary glands (including in the palate, cheek, floor of the mouth, base of the tongue and the retromolar area) (1). About one-third of diagnosed patients may have local pain or numbness or facial paralysis due to oncocytic carcinoma invading the facial nerves. The tumour is hard, inactive and has no obvious boundaries with the surrounding tissue. The skin on the surface of the oncocytic carcinoma may change colour and shrink (2). Oncocytic tumours may have a history of rapid growth from recent long-standing oncocytic carcinomas (3–5). There are also reports of oncocytic carcinomas in the nasal cavity, the paranasal sinuses (i.e. the ethmoid sinus, maxillary sinus and lacrimal sac), the bronchus, thyroid, parathyroid, kidney, ovary, upper mediastinum, lung and breast (6–9). Scattered case reports show oncocytic carcinomas occurring in the orbital area are extremely rare, and there are no reports available on oncocytic carcinomas occurring in the cranio-orbital area (10–18).

## Case Report

**Figure 1:**
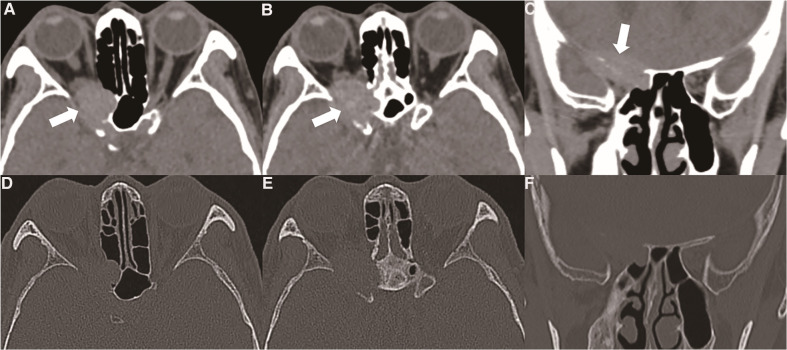
(**A**, **B**) orbital CT showed that the oncocytic carcinoma affects intracranial structures through the supraorbital fissure, accompanied by bone destruction (thick white arrow). (**C**) Oncocytic carcinoma destroyed the skull base bone and involved the dural structure (thick white arrow). (**D, E, F**) Orbital CT bone window showed the bone of the skull base and the outer orbital wall were absorbed or destroyed by the tumour.

The patient was a 61-year-old Asian male. The visual acuity of his right eye gradually worsened for almost one year before surgery. There was no swelling, pain or abnormality of movement in his right eye, but there was a defect of the visual field. The patient was first diagnosed with decreased vision in an ophthalmic clinic. The orbital computed tomography (CT) results visualised the tumour in the right posterior upper orbital space as fast growing and erosive in the cranio-orbital area, eroding and destroying the bone and protruding into the brain through the supraorbital fissure (Figure 1). The orbital enhanced magnetic resonance imaging (MRI) findings detected a solid lesion outside the muscular cone of the right superior posterior orbit.

**Figure 2:**
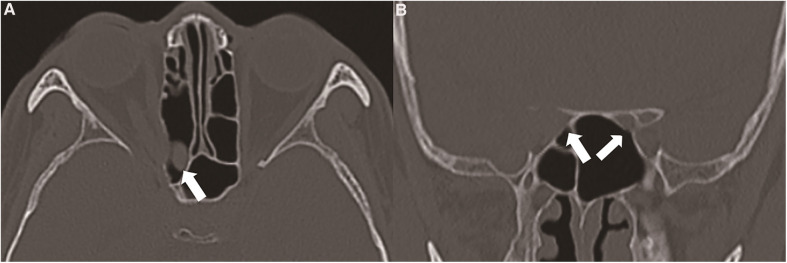
(**A**) the suspected tumour originated in the sphenoid sinus or invaded the sphenoid sinus from the cranio-orbital area (thick white arrow). (**B**) The tumour destroyed the right optic canal and involved the right optic nerve, and the side wall of the sphenoid sinus remained intact (thin white arrow). The left optic canal was intact (thin white arrow).

A transfrontal orbital craniotomy was performed. The frontal bone flap and brow bone were removed with a milling cutter. The great wings of the sphenoid bone, the bone of the skull base and the bone of the lateral orbital wall were extensively involved, and the tumour had abundant blood supply, soft texture, no complete encapsulation and some necrosis. The bone of the lateral orbital wall was removed to reach the infraorbital fissure, the sphenoid bone was cut to reach the superior orbital fissure, the bone of the supraorbital wall was removed and the orbit was fully decompressed. The right optic canal was opened, and the sphenoid was opened inward toward the sinus. The bilateral optic canal bones were intact without tumour invasion, and the suspected eosinophilic carcinoma in the sphenoid sinus was removed (Figure 2). The dura mater of the skull base and the outer part of the orbital periosteum were also removed as they were thickened and tough. The operation exhibited difficulty in that only one fronto-orbital craniotomy method was used to deal with the tumours in the sphenoid sinus, orbit and skull base dura mater simultaneously. In reviewing the case, it was seen that the oncocytic carcinoma involved the right cranio-orbital area.

**Figure 3:**
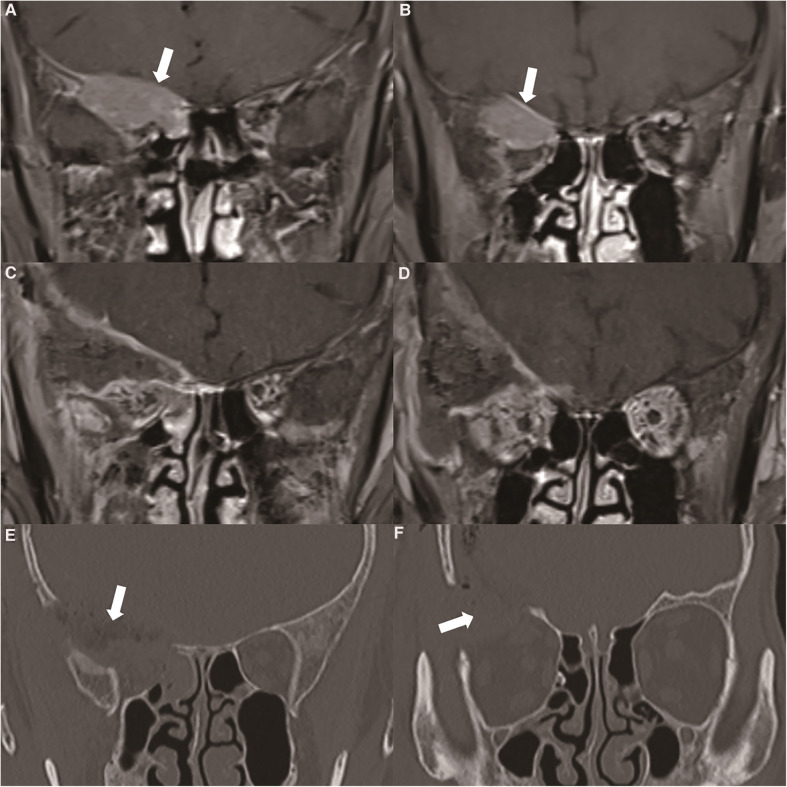
(**A**) orbital MRI showed that the tumour broke through the skull base and involved the intracranial structure (thick white arrow). (**B**) Enhanced MRI showed enhanced performance of the dura mater located at the base of the skull (thick white arrow). (**C, D**) Postoperative enhanced MRI showed complete resection of the tumour. (**E, F**) We performed a frontal orbital craniotomy to remove the tumours in the cranio-orbital area, and simultaneously performed bone removal of the skull base and the lateral wall of the orbit. Postoperative CT showed that the bone involved in the tumour was completely removed (thick white arrow).

**Figure 4:**
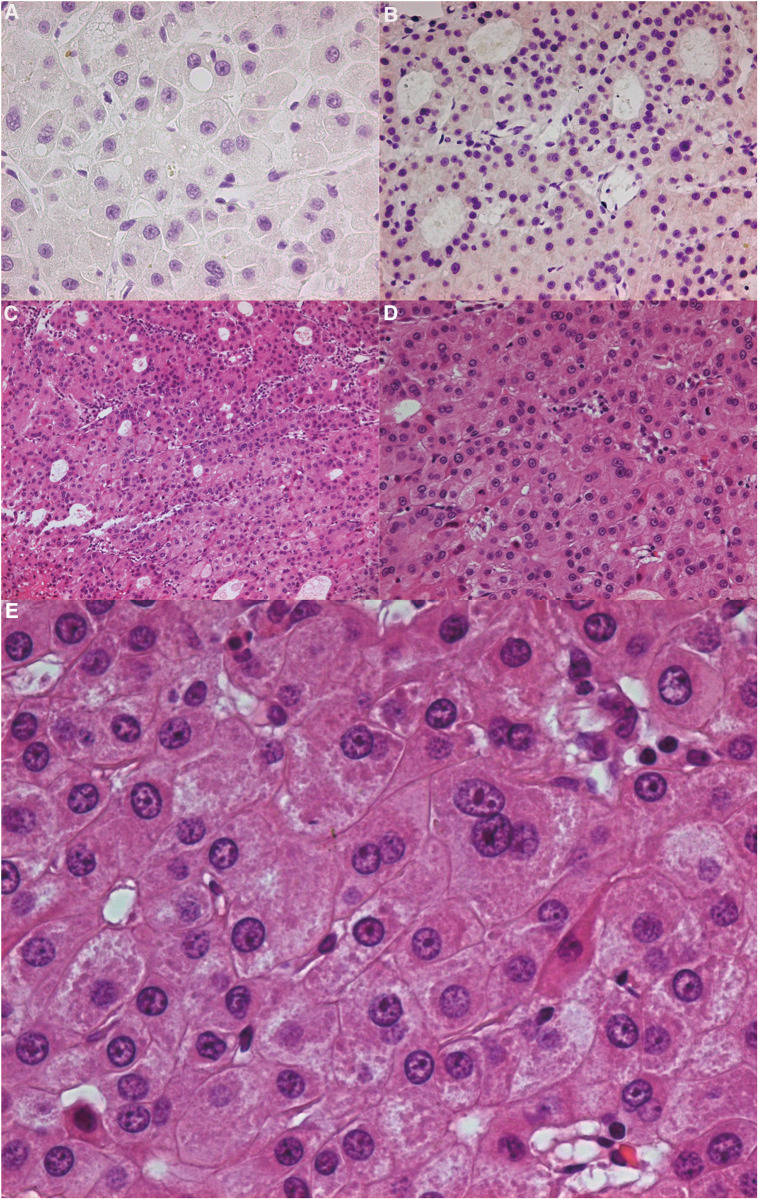
(**A**) immunohistochemistry CK7 (−). (**B**) Immunohistochemistry P63 (−). (**C**) (HE 100×): Tumour cells were usually arranged in slices, beams or alveolar structures. Occasionally, there was extensive duct differentiation in tumours. The tumour had grown invasively, and perineural and vascular invasion were common. (**D**) (HE 200×), (**E**) (HE 400×): It could be seen that the tumour cells of eosinophilic carcinoma were large, round or polygonal, and the cytoplasm was finely granular. It was more pleomorphic than an eosinophilic adenoma. The nucleus was located in the centre of the cell and often had large and irregular nucleoli. HE: Hematoxylin-Eosin staining.

The intracranial dural structure had a natural barrier effect on this kind of tumour. According to the orbital MRI, the tumour involved the dura mater of the skull base, but it did not break through the dura mater. It only caused the enhancement of the dura mater. Note that this kind of tumour must be resected in addition to the involved bone. To avoid tumour recurrence, an extensive resection of the whole sphenoid wing and lateral orbital wall was performed (Figure 3). Through postoperative pathological analysis, the immunohistochemical staining of the tumour was found to be cytokeratin (CK) 7 (−) and tumour protein p63 (−). The CK cells were marked as epithelial cells, and p63 marked various types of cells, such as squamous epithelium and myoepithelium. To help identify some morphologically similar lesions, the negative situation raised doubts regarding the origin of the tumour. The tumour cells were large, round or polygonal, and the cytoplasm was finely granular. It appeared to be more pleomorphic than the eosinophilic adenoma. The nucleus is the centre of the cell, and nucleoli are often large and irregular (Figure 4). The patient did not undergo chemotherapy after the operation, refusing because of economic factors. After extensive resection of the tumour, there was no evidence of a recurrence at the patient's 6-month follow-up.

## Discussion

Oncocytomas are common in the kidneys, parotid glands and other organs, and have corresponding diagnosis and treatment characteristics (19, 20). The case presented here was an oncocytic carcinoma located in the cranio-orbital area, which has not been found in the literature previously. Although there have been 15 reported cases of oncocytic carcinoma in the orbital area, there have been no reports of oncocytic carcinoma involving the brain (21). Oncocytic carcinomas in the orbital area generally originate from the lacrimal tissue, and most of these were located on the outside the orbit. Patients usually develop exophthalmos, making clinical discovery and early diagnosis straightforward. Like oncocytic carcinomas, patients with ocular lymphoma also commonly present with exophthalmos. Sang et al. described a case of a 6-year-old male who presented with bilateral rapid orbital swelling for 10 days, and the final diagnosis was bilateral orbital secondary non-Hodgkin's lymphoma (22). Ngoc et al. reported a case of a 74-year-old man with non-Hodgkin's lymphoma who also presented with bilateral eye proptosis (23). The final judgement of tumour type must be determined by means of biopsy.

In this case, the oncocytic carcinoma involved the deep part of the orbit, and the main body was located above the orbit. The main clinical manifestation was decreased vision with an insidious onset. The oncocytic carcinoma involved the intracranial structure through the supraorbital fissure. These situations are difficult for ophthalmologists to deal with through the transorbital surgical approach. Neurosurgeons are required to perform a craniotomy through the frontal orbital approach, removing the frontal and brow bones to completely expose the surgical area. In these cases, the oncocytic carcinoma involves the contents of the orbit, the optic nerve, the total tendon ring tissue and the dura mater of the frontal lobe. It destroys the bone of the optic canal and supraorbital fissure, the sphenoid bone and its great wings and the bone in the lateral orbital wall. This is obviously characteristic of malignant eosinophilic carcinomas. Gray et al. described the diagnostic criteria for oncocytic carcinoma as: (1) distant metastasis; (2) regional lymph node metastasis; (3) perineural, intravascular or intralymphatic invasion; and (4) common mitosis and cellular pleomorphism accompanied by extensive invasion and destruction of adjacent tissues (24).

The origin of this case of oncocytic carcinoma is worth exploring because of three aspects: (a) The patient had bone involvement in the sphenoid sinus. Part of the main body of the oncocytic carcinoma protruded into the sphenoid sinus (Figure 2). This kind of oncocytic carcinoma is known to occur in the paranasal sinus. Oncocytic carcinoma originates from the gland mucosa in the paranasal sinus and invades the orbital structure. It next invades the intracranial dural structure through the supraorbital fissure. However, in this case, the sphenoid sinus bone was basically intact, albeit thin; (b) The distant metastasis of this atypical cranio-orbital oncocytic carcinoma could not be ruled out. This kind of oncocytic carcinoma has a high degree of malignancy and is prone to distant metastasis through the lymphatic and blood systems. However, the patient was examined with positron emission CT, and no original lesions in the related organs were found; (c) The lacrimal gland structure of the patient was intact, and the main body of the oncocytic carcinoma was in the orbital apex region. This kind of oncocytic carcinoma did not originate from the lacrimal gland structure, but a primary oncocytic carcinoma in the intracranial and cranio-orbital area had not been reported. The origin of oncocytic carcinomas in the cranio-orbital area is worth discussing. The possibility of (a) and (b) still existed. In this case, postoperative enhanced MRI confirmed that the oncocytic carcinoma had been completely removed surgically. During the operation, the resection of the bones involved was extended, including the great wings of the sphenoid bone, the lateral wall outside the orbit and the lateral wall of the sphenoid sinus. The visual acuity of the patient recovered from 0.1 to 0.3, and there was no eye movement disorder. At the patient's 3-month follow-up, no recurrence of the oncocytic carcinoma was found.

## Conclusions

Oncocytic carcinomas in the cranio-orbital area are extremely rare. Because surgery involves ophthalmological and neurosurgical areas, it is difficult to completely resect the oncocytic carcinoma in a single department. Early resection is the most effective treatment for this kind of oncocytic carcinoma, and long-term follow-up and adjuvant chemoradiotherapy are beneficial. As this oncocytic carcinoma has an uncertain origin, continual systemic observation and local re-examination are needed.

## Data Availability

The original contributions presented in the study are included in the article/Supplementary Material, further inquiries can be directed to the corresponding author/s.
